# Intraoperative fluorescence molecular imaging accelerates the coming of precision surgery in China

**DOI:** 10.1007/s00259-022-05730-y

**Published:** 2022-03-01

**Authors:** Zeyu Zhang, Kunshan He, Chongwei Chi, Zhenhua Hu, Jie Tian

**Affiliations:** 1 Beijing Advanced Innovation Center for Big Data-Based Precision Medicine, School of Engineering Medicine, Beihang University, Beijing, China; 2 CAS Key Laboratory of Molecular Imaging, Beijing Key Laboratory of Molecular Imaging, The State Key Laboratory of Management and Control for Complex Systems, Institute of Automation, Chinese Academy of Sciences, Beijing, China; 3 State Key Laboratory of Computer Science and Beijing Key Lab of Human-Computer Interaction, Institute of Software, Chinese Academy of Sciences, Beijing, China; 4 University of Chinese Academy of Sciences, Beijing, China

**Keywords:** Molecular imaging, Precision surgery, Cancer surgery, Image-guided surgery, Molecular-targeted agents, Clinical translation

## Abstract

**Purpose:**

China has the largest cancer population globally. Surgery is the main choice for most solid cancer patients. Intraoperative fluorescence molecular imaging (FMI) has shown its great potential in assisting surgeons in achieving precise resection. We summarized the typical applications of intraoperative FMI and several new trends to promote the development of precision surgery.

**Methods:**

The academic database and NIH clinical trial platform were systematically evaluated. We focused on the clinical application of intraoperative FMI in China. Special emphasis was placed on a series of typical studies with new technologies or high-level evidence. The emerging strategy of combining FMI with other modalities was also discussed.

**Results:**

The clinical applications of clinically approved indocyanine green (ICG), methylene blue (MB), or fluorescein are on the rise in different surgical departments. Intraoperative FMI has achieved precise lesion detection, sentinel lymph node mapping, and lymphangiography for many cancers. Nerve imaging is also exploring to reduce iatrogenic injuries. Through different administration routes, these fluorescent imaging agents provided encouraging results in surgical navigation. Meanwhile, designing new cancer-specific fluorescent tracers is expected to be a promising trend to further improve the surgical outcome.

**Conclusions:**

Intraoperative FMI is in a rapid development in China. In-depth understanding of cancer-related molecular mechanisms is necessary to achieve precision surgery. Molecular-targeted fluorescent agents and multi-modal imaging techniques might play crucial roles in the era of precision surgery.

## Introduction

Surgery plays a vital role against the rising threat of cancer [1]. The expected goal of cancer surgery is to completely remove the cancer cells with minimum functional impairment in the least possible time [2]. To achieve this goal, high surgical precision is hugely required. Yet in the current stage, visual and tactile inspections are still the main ways for surgical decision making. These inspections are obviously experience-dependent and subjective which consequently restrain the surgical outcomes [2–4]. How to raise the surgical precision has become an important topic.

In the past centuries, various techniques have been developed to arm surgeons and the operation room. Computed tomography (CT), magnetic resonance imaging (MRI), ultrasound, and nuclear medicine imaging have become common tools for preoperative diagnosis [5]. These non-invasive imaging techniques can considerably help surgeons for surgical planning and treatment improvement [6, 7]. Nevertheless, some problems regarding surgical guidance and decision making remain unsolved. The widely applied CT and MRI have limited sensitivity and accuracy to discover subtle lesions, and are hard to provide intraoperative real-time guidance. The practicability of positron emission tomography (PET) is also limited for surgical guidance, even though PET works well to detect small lesions and metastases [5]. Besides, intraoperative CT or PET may cause the concern of radioactive safety. Ultrasonography shows a strong practicability in both diagnosis and the surgical treatment. However, its quality is dependent on the examiner’s experience and the superficial lesions (like the residues) might be occult in ultrasonography. Comprehensively speaking, an ideal surgical guidance technique would be better to have following qualities: high sensitivity, favorable resolution, adjustable vision, fast imaging speed, and non-radiative.

**Fig. 1:**
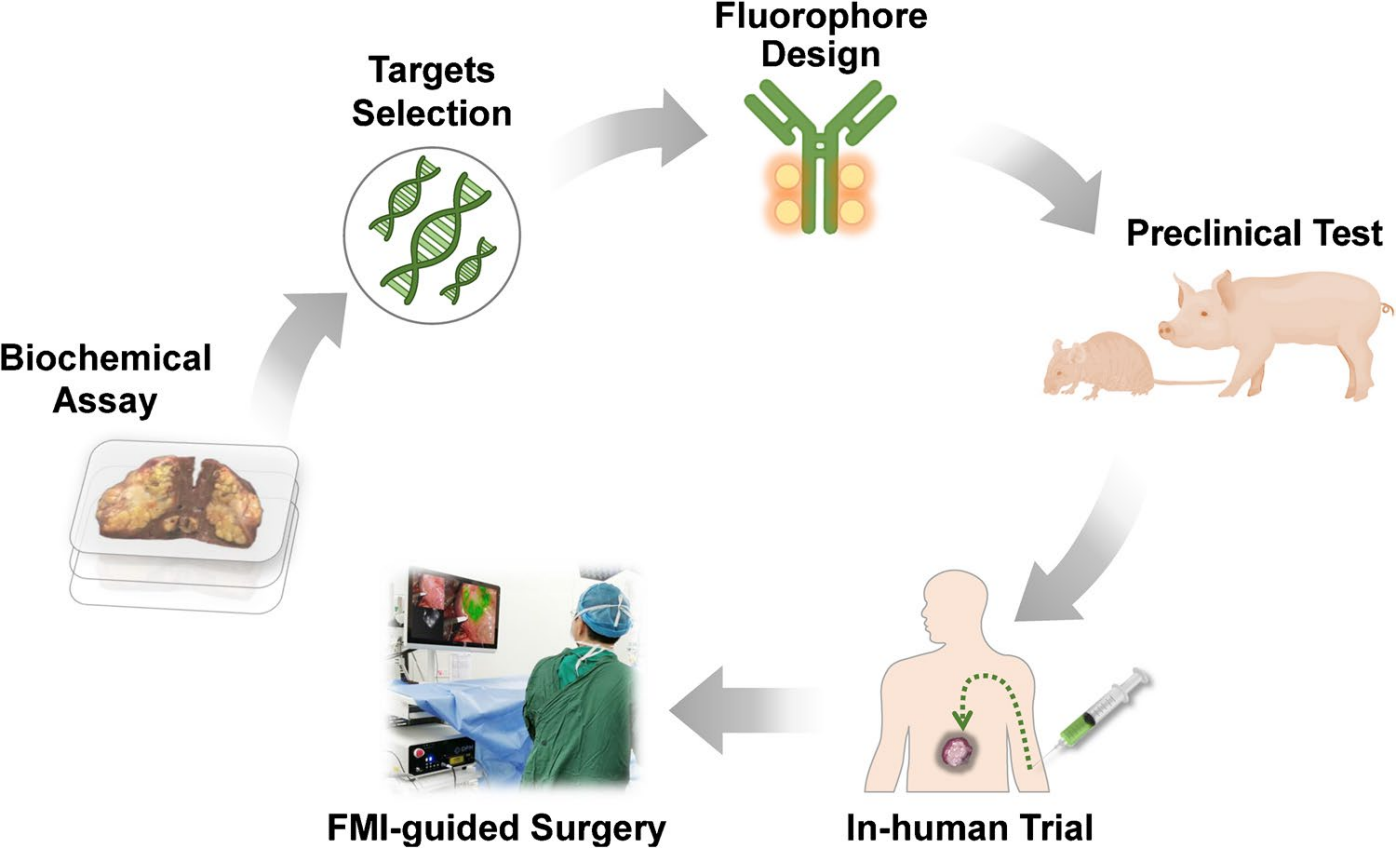
The crucial steps of the clinical translation of novel intraoperative FMI techniques

After several decades of evolution, the fluorescence molecular imaging (FMI) has now become an attractive modality to handle surgical problems regarding cancer [8]. With administration of fluorescent agents, FMI helps surgeons to make surgical decisions in a manner of real-time visualization (Fig. 1). Compared with the abovementioned CT, MRI, PET, or ultrasonography, FMI can reach picomolar sensitivity and micrometer spatial resolution and could acquire hundreds of images per second. The fluorescence images are intuitive to observers, which is beneficial to reduce the cost of operative training. Moreover, the dainty FMI imagers can be easily utilized in hands and transferred between different departments [4]. Thanks to these advantages, FMI shows a great potential in various surgical applications. From the 2000s, the FMI-guided surgery has been verified safe and effective in many applications [3, 9]. An increasing number of surgeons are getting familiar with this promising technique. So far, a series of intraoperative FMI devices have been registered for clinical use in the fast-growing market, which can serve endoscopy, laparoscopic and open surgery.

Imaging agent as another key element, its usage directly influences the application and the efficacy of FMI. Widely different from the device market, only few fluorescent imaging agents own the clinical approval up to now, including fluorescein (FS), methylene blue (MB), indocyanine green (ICG), and 5-aminolevulinic acid (5-ALA) [10].

Among them, ICG becomes the first choice in most clinical surgeries. The near-infrared (NIR) fluorescence generated by ICG has strong imaging contrast, good resolution, and ideal penetration in vivo. However, these 4 approved fluorescent imaging agents do not have the capability of targeting cancer cells. Non-ideal sensitivity or false-positive has been widely reported in diverse trials. Current achievements are still far from the expected high-precision cancer surgery. Recent progresses originated from Europe and the USA bring a new trend to develop molecular targeted fluorescent imaging agents [11]. A new folate receptor-alpha targeting agent “OTL38” has been approved by FDA in November 2021 [12]. This would be a milestone for marching in the era of precision surgery.

As for China, the world’s largest cancer population leads to an increasing requirement of precision surgery. Because of late diagnosis, more resectable cancer cases may have a higher stage in China. Trials involving these patients could help better understand how much prognosis could be improved by FMI-guided surgery. China also has special molecular profiles in many cancers. For instance, approximately 61% lung adenocarcinoma patients in China have an EGFR mutation. In contrast, the EGFR mutation rate was reported only 11% in US and European patients of lung adenocarcinoma [13, 14]. The clinical data from Chinese cohorts is valuable to complete the consensus guidelines and promotes FMI-guided surgery. In the following, we would like to briefly review the recent Chinese clinical achievements by using intraoperative FMI, including some new experiences in the second near-infrared wavelength window (NIR-II, 1,000 ~ 1,700 nm). We hope that this paper may help surgeons further explore the potential of FMI in the clinic and improve cancer surgical outcome.

## Intraoperative visualization of malignancies

Complete resection is the expected goal of cancer surgery. Surgical margin delineation is therefore one of the most important decisions during surgery. FMI, which expands the eye inspection, shows a great potential to reveal more details of cancer. The surgical margins can be clearly visualized by FMI to improve surgical decisions. Although the cancer specificity is not ideal enough, FS, MB, and ICG provide highly practical ways to perform high-precision surgery.

**Fig. 2:**
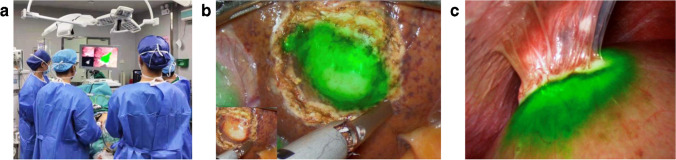
Typical intraoperative FMI of malignancies. **a** FMI “expands” the visions of the surgeons during surgery. **b** Visualization of well-differentiated HCC under the fluorescent laparoscopic view. **c** A liver metastasis of colorectal cancer was delineated fluorescently. The images were acquired using DPM-III-01 system (Zhuhai Dipu Medical Technology Co., Ltd.) intraoperatively

Hepatic cancer stays a severe threat to China. Nearly 50% of the global hepatic cancer patients occur in China [15]. Many of these Chinese patients have a poor liver function and an obvious demand for precision surgery [16]. In the last decade, by using ICG, the value of intraoperative FMI has been well comprehended in hepatic surgery. The usage of ICG is recommended to be adjusted according to the patient’s liver function [17, 18]. At least 2 days prior to surgery is recommended for ICG injection to image the lesion in poorly functional liver [19]. With the aid of FMI, high-difficulty hepatic surgeries can become much safer in implementation [20–22]. For example, the highly difficult laparoscopic anatomical resection of hepatic segment VII could be successfully performed by the precise margin visualization [23]. A clear reveal of the cancer margin directly helps reach the maximum liver function preservation (Fig. 2), especially for the patients with removable hepatic recurrences [24, 25]. Another method to visualize hepatic cancer margin was using intraoperative ICG injection. The cancer area would be rapidly distinguished in a non-fluorescent manner [26]. In this study, Zhang et al. also gave the suggestions of administration routes based on their observations. A retrospective analysis conducted by Dai and colleagues demonstrated that FMI-guided resection could achieve a wider negative margin, and therefore lead to a better prognosis compared with the traditional surgery [27]. With its promising performance, FMI-guided robotic surgery may stand for a future trend of precision treatment. Li et al. have preliminarily studied the efficacy of this novel robotic surgery on 23 patients with focal nodal hyperplasia [28].

Metastasis removal is another expectation for cancer surgery. The hepatic metastasis surgery using ICG FMI has already been included in the consensus guidelines [29]. A possible hindrance to ICG-guided hepatic metastasis surgery is the false-positive. As mentioned above, many hepatic cancer patients in China have the problem of liver cirrhosis and false-positive FMI results are usually observed. Intraoperative use of ICG may partially overcome this problem by negative-staining the cancer lesions [26], whereas the sensitivity and specificity may be affected. Recent progresses showed new FMI applications to visualize cancer in three-dimension (3D) [30, 31]. The 3D imaging approach proposed by Fang and colleagues can help surgeons delineate the surgical area with higher accuracy. Consequently, the patients could have well-preserved liver function and shortened hospitalization. Using the 3D FMI, surgeons can assess the liver volume to be resected during surgery. High consistency between the resected volume and the surgical plan was achieved in their studies [32, 33]. Precise hepatic resection therefore could be expected to excellently preserve the functional liver volume. In China, liver function preservation is a key consideration of hepatic surgery because of the hepatitis virus infection. This practical intraoperative 3D FMI may become a widely accepted approach in the future. Another study performed by He et al. exhibited the merits of ICG FMI in surgery of colorectal liver metastases [34]. In their research, ICG FMI localized more lesions than the traditional inspection (3.03 ± 1.58 versus 2.28 ± 1.35, *P* = 0.045). Importantly, the 1-year cancer recurrence rate was significantly reduced by using intraoperative FMI (ICG group 19% versus 47% in control group, *P* = 0.017).

Pulmonary surgery is a great developing area for ICG FMI as well [35–37]. It has been verified that 24 h preoperative injection of 5.0 mg/kg ICG through the peripheral vein can obtain a good performance to image and resect the pulmonary nodules. The surgeries were usually conducted with thoracoscopic observation. Guided by ICG FMI, a higher precision could be achieved in pulmonary nodule detection and resection. In a study with 36 enrolled patients accomplished Tian, Wang, and colleagues, the FMI located nine occult lesions that were missed by preoperative imaging [38]. This finding is believed valuable to facilitate the surgical decisions. Their final data showed a sensitivity of 88.7% and a positive predictive value of 92.6%. The surgical area could also be visualized in a negative-stained manner. Intraoperative injections of 0.2 ~ 0.6 mg/kg ICG through the peripheral venous catheter have been evaluated in different trials [39, 40]. The surgical area would remain non-fluorescent and surrounded by the obviously fluorescent normal pulmonary tissues.

Fluorescein-guided FMI surgery has also been studied, especially for glioma surgery. Because of the disruption of blood–brain barrier (BBB), the non-specific fluorescein was able to accumulate and illuminate the glioma [41, 42]. Given that a low-dose (1–2 mg/kg) fluorescein has been proved capable of guiding the cerebral cancer surgery [42, 43], this BBB disruption–dependent mechanism makes this fluorescein FMI method more suitable to high-grade gliomas. The sensitivity for low-grade lesions might be lower than expected [41]. A study involving 220 patients with spinal gliomas (by Sun et al.) provided an obvious evidence that the fluorescein FMI could improve the resection rate of the MRI-enhanced lesions [44]. This intraoperative method was also proved a strong feasibility to image astrocytoma and the brain metastasis from other malignancies [45, 46]. The potential of fluorescein FMI to raise the surgical outcomes in pediatric patients with brainstem gliomas was also explored by Xue et al. [47]. A total glioma removal was accomplished on 9 patients, while another 3 cases had a mean resection rate of 93.7%. Yet we should notice that the non-specific fluorescein may cause false-negative margin to the high-grade glioma [48]. Expanded careful exploration may be needed to achieve the precision resection without residue.

Compared with ICG and fluorescein, 5-ALA and MB are used less frequently in China so far. A study (by Chan et al.) with 16 enrolled glioma patients showed that the orally taken 5-ALA (20 mg/kg·m^2^) could specifically illuminate the glioma and aid in achieving microscopic total resection [49]. To the superficially located lesions, 5-ALA coating could be a feasible alternative to oral administration. In surgeries of penile-scrotal extramammary Paget’s disease, FMI from the coated 5-ALA helped accurately delineate the surgical area [50]. Another agent, MB, is often used as a staining dye to locate the lymph nodes [51–53]. While using its fluorescent property, MB showed the feasibility to visualize the breast tumor intraoperatively; yet the effects from preoperative chemotherapy should be noticed [54].

These above trials represent the efforts made to improve surgical outcome in China. ICG applications have been widely studied, particularly in hepatic cancer. The NIR emission and the plasma-binding property also make ICG more suitable to perform in vivo cancer imaging [55]. Although the cancer specificity of these imaging agents is limited, the FMI performance could be improved by adjusting the agent administration. Moreover, combined employment of these fluorescent agents could be an effective and practical way to further amend the surgical outcome [56].

## Cancer imaging in NIR-II spectrum

In 2017–2018, ICG was confirmed capable of emitting the fluorescence within the second near-infrared wavelength window (NIR-II, 1000–1700 nm) [57–59]. This important discovery expanded the application area of ICG and provided a quick way to perform NIR-II imaging in clinic.

The first-in-human NIR-II imaging study was completed in 2020 [60]. In this 23 liver cancer patients’ study, Hu et al. verified the feasibility of intraoperative NIR-II FMI and compared the performances between NIR-II and NIR-I imaging. Notably, NIR-II imaging found 3 extra lesions that were missed by NIR-I, leading to a meaningful increase of the sensitivity (NIR-II 100% versus NIR-I 90.63%) and the accuracy (NIR-II 91.43% versus NIR-I 82.86%). This study opens the door to following clinical translations of NIR-II and multi-spectrum imaging techniques.

A NIR-II-guided glioma surgery has been reported by Shi et al. [61]. This new surgical method was performed on 41 glioma patients; the high-grade glioma detection rate was reported as 100% using ICG NIR-II imaging. Traditional white-light surgery was performed as the comparison. From their follow-up data, significant improvements can be seen in the progression-free survival (median of NIR-II group 9 months, while white-light group 7 months) and overall survival (median of NIR-II group was 19.0 months; white-light group was 15.5 months). When integrating machine learning, more diagnostic information could be extracted from the glioma NIR-II results [62]. Shen et al. designed deep convolutional neural networks to automatically analyze the NIR-II glioma in situ images. The machine learning method had a sensitivity of 93.8% (versus 82.0% by neurosurgeons) in glioma detection, and could rapidly predict the cancer grade and even the Ki-67 expression. This interesting study exhibited the potential of machine learning for FMI and showed an intraoperative pattern to implement.

ICG NIR-II imaging has been employed as well in nephron-sparing surgery [63]. Determining the margin of renal carcinoma is a challenging problem. Cao et al. used ICG to delineate the lesion’s margin by NIR-II negative fluorescence. After the injection of ICG, the normal renal parenchyma was illuminated in NIR-II imaging while the lesion area remained dark. The contrast ratio between the lesion and the normal tissue could reach over 4.8, which was distinguishable and ideal for negative-staining imaging. Complete cancer resection was achieved in all the enrolled patients.

These recent clinical observations of NIR-II showed notable improvements on imaging performance and the surgical outcome. Benefiting from high spatial resolution, strong imaging contrast, and enhanced penetration, NIR-II with ICG showed better performance in small lesion detection and margin determination [55]. The integration of NIR-II imaging and machine learning is also attractive. A compact and economical system is beneficial to promote the use of NIR-II imaging. And multi-center involved randomized clinical trials are expected to better verify the surgical outcomes between NIR-II and traditional approaches. Briefly, more clinical applications regarding NIR-II should be encouraged in clinic.

## Lymphatic FMI imaging

Dissection of lymph nodes is important for many cancer surgeries, such as breast cancer, gastric cancer, and colorectal cancer [64–66]. The main goal is to remove the possible cancer metastases in the lymphatic system [67, 68]. Locating lymph nodes is often difficult in surgery because of the complicated connection between lymphatic vessels [69]. FMI-guided lymphadenectomy can reduce the non-compliance rate and lead to a better prognosis [70, 71]. In China, the FMI-guided lymphadenectomy has been proved worth spreading in gastric cancer, which has a specifically high incurrence rate in Eastern Asia [72].

**Fig. 3:**
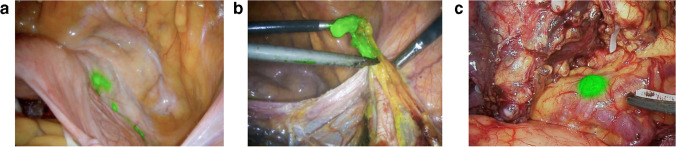
FMI-guided lymphadenectomy in cervical cancer (**a, b**) and gastric cancer (**c**). The images were acquired using DPM-III-01 system (Zhuhai Dipu Medical Technology Co., Ltd.) intraoperatively

A double-armed randomized clinical trial involving 266 patients was conducted by Huang and colleagues, to study the feasibility and efficacy of ICG FMI–guided laparoscopic lymphadenectomy [73]. The patients in the experimental group received an endoscopic peritumoral injection of ICG to the submucosa 1 day before surgery. When laparoscopic D2 lymphadenectomy was performed, ICG fluorescence could retrieve 50.5 ± 15.9 lymph nodes, while the control group can retrieve 42.0 ± 10.3 nodes. Thus, the intraoperative ICG FMI was demonstrated efficient to improve gastric D2 lymphadenectomy without increased complications (Fig. 3c). The same surgical team further compared the performance of sub-mucosal and sub-serosal injection of ICG [74]. A total of 259 patients were enrolled in this per-protocol analysis. The clinical results indicated a comparable performance between sub-mucosal and sub-serosal injection (lymph node noncompliance rate 32.3% versus 33.3%, *P* = 0.860). Yet the sub-mucosal method needs a preoperative gastroscopy, which may cause mental burden and extra economic cost. Therefore, the sub-serosal injection is more recommended to perform ICG FMI–guided D2 lymphadenectomy. In an analysis by Huang et al., the improved D2 lymphadenectomy method was proved valuable for advanced gastric cancer with poor outcome to neoadjuvant chemotherapy [75]. A total of 184 matched patients were included and divided into two groups equally. Clinical observation showed that the blood loss in ICG FMI group was less than the non-ICG group. And the D2 dissection lymph nodes in ICG FMI group (39.6 ± 13.2) were significantly higher than the non-ICG group (30.8 ± 11.8). ICG FMI–guided D2 lymphadenectomy should be performed routinely for the patients with advanced gastric cancer. To evaluate the feasibility of imaging metastatic lymph nodes using ICG, Zhong et al. performed a 514 patients enrolled study [76]. The ICG FMI had a sensitivity of 86.8% to the metastatic lymph nodes, while the negative predictive value was 92.2% to the non-fluorescent nodes. The sensitivity and negative predictive value were also found related to the cancer stage. For cT1 ~ cT2 patients, both sensitivity and negative predictive value reached 100%. To the cT3 ~ cT4a patients, the sensitivity was reduced but still over 80%. These findings could aid surgeons to perform personalized lymphadenectomy based on the cancer stage.

In thoracic esophageal cancer surgery, ICG FMI–guided lymphadenectomy exhibited ideal performance (by Shan and colleagues) [77]. A four-quadrant endoscopic injection was used after anesthetization. In all the 84 involved patients, the average number of regionally dissected lymph nodes was 25.68 ± 12.00. The stained lymph nodes showed a clear mapping for dissection, but the efficacy to identify metastasis-positive nodes has not been explored yet.

These randomized clinical trials have provided high-level evidence of the FMI performance in lymphatic imaging. ICG presents a high effectiveness to visualize the regional lymph nodes and aid lymphadenectomy. However, the goal to specifically identify the metastatic nodes remains unachieved using ICG. Employing newly designed molecular-targeted imaging agents might overcome this problem [78], and multi-spectrum imaging is also believed as a feasible approach [56].

## Intraoperative FMI aids reduce iatrogenic injury

Iatrogenic injury is catching attention nowadays. A series of Chinese groups have shared their experience to rduce iatrogenic injury by utilizing intraoperative FMI.

Biliary injury in hepatic surgery may cause severe complication and lead to a bad prognosis. ICG FMI showed the feasibility to visualize biliary duct intraoperatively and reduce the incurrence of biliary injury. Especially for the patients with extensive dense adhesions around the hepatoduodenal ligament, FMI can provide important assistance to avoid biliary injury [79]. Su, Li, and colleagues implemented this method through an injection of low-dose ICG (2.5 mg) in surgical procedures. Further study also demonstrated that the biliary duct imaging performance could be optimized with 10 mg ICG injections 10 to 12 h prior to surgery [80]. ICG FMI can also play a role in living donor liver transplantation [81]. By visualizing the surgical margin and the biliary branches in real time, the hepatic function of the donor could be preserved as much as possible. This method has been proved safe and feasible by Li et al. in pediatric liver transplantation, which guided in situ reduction of liver segment and effectively preserved liver function of the donor [82]. If more high-level evidence supported, ICG FMI may become an efficient alternative to the complicated intraoperative cholangiography in specific hepatic surgeries. Analogous preservation demand exists in thoracic surgery. The FMI method using ICG has been reported (by Yang et al.) to intraoperatively image and preserve the thoracic duct [83]. Subcutaneously injected ICG (0.2 mg/kg) into the bilateral inguinal region (0.5 h prior to surgery), any fistula sites could be clearly found and repaired with FMI observation. Similar use of ICG or fluorescein also performed a precision FMI angiography in the high-precision-demanded cephalic and eye-sparing surgery [84, 85].

The application of FMI has been expanded to the emerging nerve imaging in recent years. Most nerves are thin and indistinguishable by human eye observation [86, 87]. Complications (like palmar hyperhidrosis) are common because of the iatrogenic injury to the vulnerable nerves [88, 89]. In 2016, ICG FMI has been reported (by Wang and colleagues) as an efficient method to visualize and help preserve the nerves during thoracoscopic surgeries [90]. He et al. further validated the ICG usage on rabbits and a 15-patient cohort [91]. Their findings indicated that preoperative injection (24 h prior to surgery) of 5 mg/kg ICG could achieve an ideal performance (imaging contrast ratio 3.26 ± 0.57) of thoracic sympathetic nerve imaging. The retrospective data from 142 patients verified that the ICG nerve imaging is safe and practical to help reduce complication rates [92]. There is also a noticeable ongoing trial aiming at preserving pelvic nerves in radical hysterectomy of cervical cancer [93]. Similar ICG FMI strategy was introduced in the registration (NCT04224467, recruiting in Nanfang Hospital). Although surgical outcomes have been achieved, the mechanism of ICG intake by nerves is not crystallized yet. It is worth believing that FMI might become more effective in nerve preservation when the mechanism can be fully understood.

## Multi-modal imaging promotes precision surgery

So far in China, cancer targeted fluorescent agents are rarely reported in the clinic. Applying ICG, FS, MB, and 5-ALA has inevitable false-negative and false-positive, which may affect the surgical accuracy. The successful translations of molecular-targeting fluorescent agent like OTL38 reflect the future trend for precision surgery. But the route of developing promising molecular-targeting FMI agents is long, and strong multidisciplinary collaboration is indispensable. In the process of producing new molecular-targeting fluorescent agents, multi-modal imaging has a great worth to elevate the surgical accuracy [94, 95].

PET often represents the highest sensitivity of preoperative cancer diagnosis [96, 97]. Different from FMI, the innovative PET tracers could be translated into the clinic efficiently [98, 99]. Many cancer-specific PET tracers have been proposed in China and contributed to surgical planning [100, 101]. The whole-body PET scan could achieve a better staging and therefore lead to precise clinical decisions [97, 102, 103]. Clinical innovations with newly designed radioactive tracers can be found in detection of hepatic cancer [100], pulmonary cancer [104, 105], glioma [106], prostate cancer [107–110], melanoma [111], lymphoma [112], etc. These new PET tracers have significantly improved the sensitivity and specificity for cancer detection. However, the impressive performance of PET can hardly be replicated in surgery. In many times, the high-quality diagnostic images may only have a limited value to surgery.

From 2009, the emerging Cerenkov luminescence imaging (CLI) provides a way to perform optical imaging using PET tracers [113, 114]. CLI makes the β + radiotracers become dual-modal imaging agents with high specificity to cancer, and it has been proved safe to perform CLI-guided surgery [115–117]. The main concern about CLI for clinical application is the low intensity of the luminescence [118, 119]. Endoscopic CLI (ECLI) was accordingly proposed in 2012 by Cheng and colleagues [120]. The cavities of human body provide ideal environments to acquire the CLI signals. Wu, Wang, Chen, and colleagues described the first human results of ECLI [121]. Four patients with rectal cancer were involved in this study. The lesions were detected after ^18^F-FDG injection, and the intraoperative ECLI observation is highly consistent with the preoperative PET diagnosis. Their work demonstrated the feasibility of cancer surgery guided by PET-optical dual modal molecular imaging. Nevertheless, the optical signal emitted from PET tracer is quite weak [122–124]. For ECLI, over 90% of the luminescence was measured to be attenuated during the transmission in the clinical endoscope [125]. Hence, a long exposure time was needed to obtain high-quality results, which might hinder the surgical process [126, 127]. Recently, intraoperative ex vivo CLI has become an emerging way for cancer margin detection [128–130]. The positive margins could be visualized intraoperatively by ex vivo CLI imaging. Meanwhile, multidisciplinary efforts have been done in China to discover novel methods to strengthen the performance of CLI [118, 123, 124], whereas these new methods are still in preclinical stages. It is worth believing that the rapid upgradation of PET tracers and imaging devices could make CLI become a powerful tool for precision surgery in the future.

Another novel dual-modal imaging strategy showed the great worth of combining PET and FMI [131]. Li et al. designed a new tracer ^68^ Ga-IRDye800CW-BBN targeting gastrin-releasing peptide receptor (GRPR) that is overexpressed in glioblastoma multiforme. This tracer owned the capability to perform both preoperative PET and intraoperative FMI. The results from 42 foci showed that the ^68^ Ga-IRDye800CW-BBN-guided surgery had a sensitivity of 93.9% and specificity of 100%. With the help of the high-specificity FMI detection, maximum safe resection is easier to achieve and 82.76% of the enrolled patients received a complete resection [132]. This PET-FMI strategy might be a valuable trial to build up the connection between molecular-specific diagnosis and surgery. Combining the results from PET and FMI, the clinical decision and the treatment outcome are believed to be hugely improved.

Furthermore, surgical observations suggest the complementation between intraoperative ultrasonography and FMI. Ultrasonography has a good performance to detect deep lesions, while FMI can visualize the surgical margin clearly and reveal small occult lesions [133]. A combination of these two intraoperative techniques could have an expanded vision and higher imaging precision [133, 134]. This combined strategy has aided to improve the R0 resection rate in China. Intraoperative ultrasonography can also guide ICG injection in laparoscopic surgeries for different purposes. A single-center study in 2020 shared the experiences of using intraoperative ultrasonography combing with FMI, to achieve precise hepatic segmentectomy, hemi-hepatectomy, and multi-segmentectomy [135].

## The coming future of precision surgery in China

**Fig. 4:**
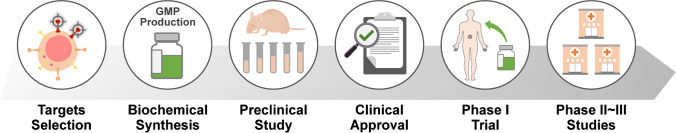
The major steps of developing molecular-targeting fluorescent agents for clinical application

The population with newly diagnosed cancer is still growing in China. There is an urgent need of precision surgery. We can foresee that the application of intraoperative FMI would be rapidly expanded [93, 136]. So far, the clinical value of non-specific fluorophores has been widely explored. Some promising results like nerve imaging have also been studied in the clinic [93]. Nevertheless, the accuracy of malignancy imaging remains unsatisfied. Lacking molecular-targeting fluorescent agents restrains the progress of precision surgery. Analytical data showed that there have been 39 new molecular-targeting fluorescent agents reported in the clinic globally (until February 2020) [137]. Antibody-dye conjugations (e.g., bevacizumab-IRDye800CW) and specially designed small molecules (e.g., OTL38) exhibited promising performances in surgical navigation [138, 139]. Yet only few of these novel fluorescent agents were relevant with Chinese groups [137]. Comprehensive efforts from basic research to clinic are required to build up an innovative environment in China for the coming era of precision surgery. From the successful translation of the new cancer-specific fluorescent agents, the essential procedures can be summarized as follows (Fig. 4): (1) determining specific bio-targets according to cancer types, (2) synthesizing the molecular-targeting fluorescent agents in compliance with good manufacturing practice (GMP) regulation, (3) testing the developed agents with living animals, (4) applying for approval of clinical trial when the agents passed preclinical tests, (5) safety and effectiveness study in patients of a small cohort, (6) further clinical study with expanded cohorts.

As mentioned above, many cancers (e.g., pulmonary cancer) in China have a special molecular profile. The existing innovative agents from abroad may not be directly suitable for Chinese cancer patients. Thus, we strongly encourage to research new surgical solution based on the characteristics of Chinese local patients. Closer collaboration between clinicians and scientists is necessary. Participation from medicine industry is also needed. The industry of innovative medicine is growing fast, and has a bright future in China [140]. Those developing companies could provide solid foundations to study novel molecular-targeting fluorescent agents.

Another notable challenge for precision cancer surgery is the heterogeneity. The existing high-level researches have shown us the complicated cellular and molecular mechanisms during the cancer progression [141–143]. These analyses pointed out the necessities to research multi-molecular-targeting imaging. A recent study exhibited the feasibility and promising performance of FMI to intraoperatively visualize the cancer heterogeneity. Chen et al. applied multiplexed FMI to improve the diagnostic accuracy of Barrett’s neoplasia [144]. A total of 22 patients were involved in this feasibility trial. The statistical analyses showed that the sensitivity and the specificity were 92% and 89%, respectively. We believe that this impressive research may open the door to image multi-biomarker in surgery, while the potential of multi-modal surgical navigation should be further explored as well.

## Conclusion

To reach the goal of precision surgery, a series of technical efforts have been made globally. FMI has contributed to improving various surgeries in China, and the application of NIR-II fluorescence may become a new trend. However, lacking cancer-targeted agents is still a great obstacle to achieve precision surgery. We hope the impressive progress of anti-cancer drug innovation could boost the development of molecular targeted tracers in China. A closer cooperation between clinicians and scientists is also strongly recommended to accelerate the progress towards the new era of precision surgery.
